# Development and Evaluation of Low‐Fat Fish and Chicken Nuggets Fortified With Date Seed Powder and Quinoa Flour as Agricultural Dietary Fiber Sources

**DOI:** 10.1002/fsn3.4749

**Published:** 2025-03-27

**Authors:** Shahab Naghdi, Masoud Rezaei, Mahboobeh Kashiri, Fatemeh Rezaei, Serva Naseri, Hossein Nourani, Zahra Khakpour

**Affiliations:** ^1^ Seafood Processing Department, Marine Sciences Faculty Tarbiat Modares University Noor Iran; ^2^ Faculty of Food Science and Technology Gorgan University of Agricultural Sciences and Natural Resources Gorgan Iran

**Keywords:** cooking yield, date seed powder, fiber‐enriched nuggets, functional properties, quinoa flour

## Abstract

In recent years, high‐oil‐content fried products, such as nuggets, have posed a significant challenge and concern for consumers, leading to increased interest in fiber‐enriched meat alternatives that offer specific functional properties and health benefits. This study investigated the incorporation of quinoa flour and date seed powder as fiber sources into chicken and fish paste formulations at a 6% concentration, with varying ratios of quinoa flour to date seed powder: 0:0 (T1), 100:0 (T2), 75:25 (T3), and 50:50 (T4). The results demonstrated that adding these ingredients markedly improved the dietary fiber content, water holding capacity, cooking yield, and pH levels of the nuggets (*p* < 0.05). Notably, the T4 treatment exhibited the lowest oil absorption (3.82 g for chicken and 5.19 g for fish per 100 g of product) among all formulations (*p* < 0.05). The fiber‐enriched nuggets exhibited noticeable differences in texture and color. Additionally, the incorporation of quinoa flour and date seed powder positively influenced the sensory attributes of the nuggets, with T3 achieving the highest overall acceptance score. This formulation was identified as the most favorable option for both chicken and fish nuggets, owing to its optimal cooking yield, high acceptance, adequate fiber content, and minimal oil absorption.

## Graphical abstract

This study examined the development of low‐fat chicken and fish nuggets by incorporating quinoa flour and date seed powder as fiber sources. Finally, the findings suggest that these fiber‐enriched nuggets offer a healthier alternative to traditional high‐oil fried products.

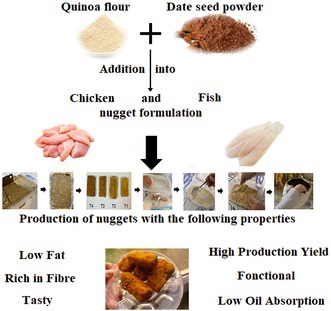

## 1 Introduction

In recent years, ready‐to‐eat food products have become increasingly popular due to changes in socio‐cultural behaviors, and global demand for high‐quality foods, especially fried foods with coatings and meat products such as various nuggets and breaded products, has increased (Azizi and Nateghi 2020; Gilbert and Khokhar 2008). On the other hand, growing concerns about the potential risks associated with consuming high‐fat foods have led the food industry to develop new and low‐fat formulations (Azizi and Nateghi 2020). Among these, edible aquatic animals, due to their significant amounts of unsaturated fatty acids and low cholesterol, various vitamins and minerals, and high nutritional value proteins, have a special place in human diets Biswas, Kandasamy, and Das (2022). Therefore, attention to consuming aquatic animals has been increasing in many communities in recent years Biswas, Kandasamy, and Das (2022); Gilbert and Khokhar (2008). According to FAO statistics in 2020, the total production of aquatic animals worldwide was 177.8 million tons (FAO 2020). Of this amount, 556,000 tons were related to total aquaculture production in Iran, of which 216,952 tons were related to the production of warm‐water fish FAO (2020). Despite the beneficial effects of seafood consumption on human health and high production of farmed aquatic animals, per capita consumption of aquatic animals in Iran in 2020 was reported to be 13.38 kg per person FAO (2020), which is less than global consumption (20.2 kg) in 2020 FAO (2020). Among the reasons for the low per capita consumption of aquatic animals, one can point to the unpleasant taste and smell, particularly noticeable in warm‐water fish Badr, Salwa, and Ahmed (2015); Liu et al. (2021). Therefore, today, to address this problem and increase the popularity of aquatic animals, various products such as fish nuggets, fish burgers, fish sausages, and more have been produced.

Chicken nuggets are also one of the ready‐to‐eat products that, with their gelatinous inner part and crispy, golden‐colored outer coating, have gained great popularity among consumers worldwide Kim et al. (2015). Breaded and fried products, including meat nuggets, absorb about 15%–20% of their weight in oil during the initial frying stage, which has caused concerns among consumers from a nutritional health perspective (Lalam et al. 2013). Consequently, these concerns have had negative impacts on the market share of such breaded and fried products (Cui et al. 2022; Shan et al. 2018). In addition, market demand for the production and consumption of meat products with special functional characteristics to increase consumer nutritional health is increasing, which has led to transformation and development in the food industry (Dehghan Nasiri et al. 2012; Kuipers et al. 2011). One of the development paths in the meat industry is the production of healthy foods with lower fat content. This development also includes creating value‐added meat products to achieve higher content of minerals, vitamins, antioxidants, or dietary fibers (Cofrades et al. 2000; Shan et al. 2018). In this regard, considering that the prevalence of gastrointestinal diseases has become a major problem in human societies, attention to the production of meat products with high fiber content has become a major concern in the food industry He et al. (2022); Yusuf, Saha, and Umar (2022). Dietary fibers include polysaccharides, oligosaccharides, lignin, or similar carbohydrates that are resistant to digestion and absorption in the human small intestine and are completely or partially fermented in the large intestine, facilitating waste elimination Dai and Chau (2017). It is worth noting that chicken and fish are considered sources with low fiber in the diet, and adding dietary fibers to these products improves their quality characteristics Buda et al. (2021). Quinoa is known as a pseudo‐cereal containing suitable protein and fiber, which can play a crucial role in food security worldwide, and its history of human consumption dates back 5000 years Ando et al. (2002). Today, quinoa is known for its high‐quality protein content and suitable amounts of lysine and methionine amino acids and can be used as a meat substitute James (2009; Wright et al. (2002). Additionally, quinoa contains significant amounts of fiber and minerals like calcium and iron Ando et al. (2002) and antioxidants like polyphenols (Repo‐Carrasco‐Valencia and Serna 2011). These features distinguish quinoa from other ordinary cereals, making it attract increasing attention worldwide. Date seed powder is another fiber source considered essential to health. Date seeds constitute 10%–15% of the weight of the date. Although date seeds are considered waste materials, they contain many valuable substances like carbohydrates, oil, dietary fiber, protein, tannins, and natural antioxidants (El‐Rahman and Al‐Mulhem 2017; Golshan Tafti, Solaimani Dahdivan, and Yasini Ardakani 2017). However, limited studies have been conducted on date seeds, and most studies have focused on the chemical composition of date seeds Ghnimi et al. (2017). Considering the above discussion and awareness of the benefits of dietary fiber and its use in the formulation of meat products, this study will use quinoa flour and date pit powder in the formulation of chicken and fish nugget dough, and the effects of these additives on the textural, functional, and sensory properties of the treatments will be investigated.

## 2 Material and Methods

### 2.1 Materials

The raw materials used in this research include chicken breast meat, quinoa flour (Mofida Company, Iran), date seed powder (Flavinea Company, Iran), silver carp fish, sunflower oil (Bahar, Iran), wheat flour (Roshd Company, Iran), egg white powder, additives and flavorings such as garlic powder, onion powder, salt, mixed dry spices (Golha Company, Iran), sodium tripolyphosphate (Karoon Chemical Company), and sodium nitrite (Karoon Chemical Company).

### 2.2 Proximal Composition

The methodologies of the Association of Official Agricultural Chemists (AOAC) were used to determine the contents of moisture (method 925.09), ashes (method 923.03), proteins (method 920.87), lipids (method 920.85), and dietary fibers (method 985.29) Echeverria et al. (2022).

### 2.3 Water Holding Capacity (WHC) and Oil Holding Capacity (OHC)

The oil holding capacities of quinoa flour and date seed powder were measured at 25°C (Durmaz and Yuksel 2021). Samples (0.25 g) were weighed into a 50‐ml test tube with a cap, and 10 mL of water and 10 mL of oil were added. The suspension was then mixed using a vortex mixer for 1 min. Subsequently, the suspension was centrifuged using a centrifuge machine (Universal320, Hettich, Germany) at a speed of 4100 rpm for 10 min. Free water and oil were removed from the suspension. WHC and OHC were calculated using the following formula:

$$\text{Water and oil holding capacities}=\text{Sample weight}/\left(\text{sample weight}-\text{sediment weight}\right)$$

### 2.4 Chicken Nugget Preparation

The chicken nugget dough was prepared according to the formulation by Echeverria and colleagues (2022) as follows: Ground chicken meat (72%) was mixed with additives and flavorings including onion powder (2%), garlic powder (0.5%), salt (1%), spices (1%), egg (3%), vegetable oil (9%), cold water (11%), and sodium tripolyphosphate (0.5%) until a uniform and homogeneous dough was obtained. This dough was considered as the base chicken nugget dough. Subsequently, quinoa flour and date seed powder (0%–6% by weight) were substituted for meat in the chicken nugget dough at different ratios (0:100, 25:75, 50:50), and the resulting mixture was molded into 5 × 2.5 cm molds. Date seed powder at ratios of 0, 1.5%, and 3% and quinoa flour at ratios of 3, 4.5, and 6% were used in the meat part of the nugget formulation. The dough ingredients included wheat flour (95%), baking powder (2.5%), red pepper (1.5%), and salt (1%). The dough preparation involved mixing dry ingredients with water (at 20°C) in a 1:2 ratio (by weight) using an electric mixer for 4–5 min. The molded samples were then packaged in zip‐lock bags and stored in a freezer at temperatures below 18°C for 24 h. Each sample was individually immersed in the dough for 30 s and then held vertically for 15 s to remove excess dough and eliminate testing errors. The surfaces of the samples were coated with medium granulated wheat flour (GolBahar, Iran) and fried in sunflower oil (at a temperature of 180°C ± 2°C for 3 min) (Bonfim et al. 2020). After cooling the samples to room temperature, the textural, functional, and sensory characteristics of samples with different treatments were evaluated. To assess the effect of the frying process on the chicken nuggets (with and without coating), the nuggets were deep‐fried in a frying machine (Black & Decker, model SL13YD, made in England) using specialized frying oil at 180°C for 3 min. The experiments related to the evaluation of freshly fried chicken nuggets were carried out immediately after cooling the samples to room temperature.

– Treatment 1: Emulsion without quinoa flour and date seed powder
– Treatment 2: Emulsion containing 6% quinoa flour
– Treatment 3: Emulsion containing 4.5% quinoa flour and 1.5% date seed powder
– Treatment 4: Emulsion containing 3% quinoa flour and 3% date seed powder

### 2.5 Fish Nugget Preparation

Silver carp fish ( *Hypophthalmichthys molitrix* ) with an average weight of approximately 1.5 kg were obtained from the local market in Mahmoodabad, immediately skinned, heads and tails removed, and gutted. The fillets were then washed and ground using a meat grinder. Fish nuggets were produced using silver carp fish, following the method by Biswas and Nanda (2023) with some modifications. The remaining steps were carried out similarly to the methods mentioned for chicken nugget production.

### 2.6 Water Holding Capacity of Fish Nuggets

The water holding capacity of fish nugget samples was measured using a centrifuge. The samples were centrifuged at 1000 rpm and at 4°C for 15 min, and the water holding capacity was calculated using the following formula (El‐Sohaimy et al. 2022).

Water holding capacity = (post‐centrifugation weight/pre‐centrifugation weight) × 100.

### 2.7 Stability of Chicken Nugget Emulsion

To assess the stability of the chicken nugget emulsion, approximately 5 g of chicken nugget dough was weighed in a test tube (*w*1). The test tube was heated at 80°C for 30 min. Then, the test tube was inverted onto a smooth paper to drain. After that, the test tube was weighed again (*w*2). The stability of the chicken nugget emulsion was calculated using the following formula (Tamsen, Shekarchizadeh, and Soltanizadeh 2018).

$$\text{Emulsion stability}=\left(\left(w1-w2\right)/\text{sample weight}\right)-1\times 100$$

### 2.8 Product Yield

The product yield was calculated as a percentage using the following formula (Shan et al. 2018).

$$\text{Product Yield}=\left(\mathrm{raw}\;\text{nugget weight}/\text{fried nugget weight}\right)\times 100$$

### 2.9 Oil Absorption Rate of Nuggets

The oil absorption rate of the samples was calculated by measuring the oil in raw and fried nuggets and subtracting the amount of fat before and after frying (Barros et al. 2018).

### 2.10 Expressible Moisture

The moisture content that can be expressed from the nuggets was determined using a modified centrifuge technique as described previously (Das et al. 2024). Approximately 5 g of finely chopped cooked nuggets were enclosed between two layers of filter paper and inserted into a 50 mL centrifuge tube. These tubes were then centrifuged at 1500 *g* with a Remi India centrifuge for 15 min, and their weights were noted before and after centrifugation. The expressible moisture content was calculated as a percentage using the formula:

$$\text{Expressible Moisture}\;\left(\%\right)=\left(\text{initial weight}-\text{final weight}\right)/\text{initial weight}\times 100$$

### 2.11 Texture Analysis of Nuggets

For texture analysis, a texture analyzer device was used to measure the texture properties of the nugget samples 3 h after deep frying at 180°C. Cubic samples with dimensions of 1 × 1 × 1 cm were separated and subjected to compression testing to determine the texture parameters under compressive testing conditions (Oppong et al. 2021).

### 2.12 Color Analysis of Nuggets

For color analysis of chicken nuggets, high‐resolution images were captured using an 8‐megapixel digital camera. The images were then analyzed using software such as Image J to separate and calculate the L*, a*, and b* factors for color assessment.

### 2.13 Sensory Analysis of Samples

**FIGURE 1:**
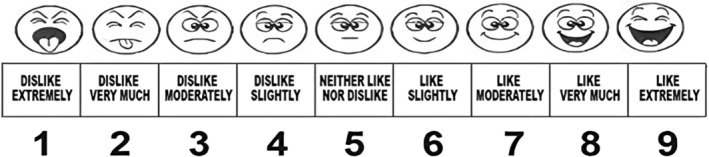
Scoring guide form in sensory evaluation of nuggets.

To evaluate the color, texture, tenderness, taste, aroma, appearance, and overall acceptance of the produced fish nuggets, a nine‐point Hedonic scale method was used. The fish nuggets were fried for 3 min at 180°C in sunflower oil and evaluated by 10 assessors, including six males and six females from Tarbiat Modares University. Assessors rated each sensory attribute (taste, color, texture, chewability, and overall acceptance) on a scale from 1 (disliked extremely) to 9 (liked extremely) (Figure 1) Das, Rajkumar, and Verma (2015).

### 2.14 Human Subjects

The study was reviewed and approved by Tarbiat Modares University IRB and informed consent was obtained from each subject prior to their participation in the study.

### 2.15 Statistical Analyses

Statistical analyses were performed using SPSS ver. 22.0. One‐way analysis of variance (ANOVA) and Duncan's multiple range test were utilized to determine significant differences between the variables. Differences were considered significant at a *p*‐value of < 0.05. The results were expressed as a mean value of three replicates ± SD (*n* = 3).

## 3 Results and Discussion

### 3.1 Approximate Composition and Functional Properties of Flours

The approximate composition of date seed powder and quinoa flour, including moisture, protein, fat, ash, and fiber, is presented in Table 1. The data obtained from this section of the research indicate its alignment with the results of other researchers; Aluwi, Murphy, and Ganjyal (2017) examined the approximate analysis of different quinoa species, reporting moisture levels between 4.10% and 33.9%, fat between 5.08% and 6.50%, protein between 6.15% and 13%, and ash between 3.38% and 3.95%. Additionally, an independent report stated that quinoa flour contains 4% fiber. Numerous studies have been conducted on the composition of date seeds in different parts of the world. Golshan Tafti, Solaimani Dahdivan, and Yasini Ardakani (2017) investigated the approximate analysis of various date species, reporting moisture levels between 3.10% and 14.3%, fat between 0.13% and 2.05%, protein between 0.87% and 2.29%, and ash between 1.35% and 0.89%. In another study, Ghnimi et al. (2017) reported the fiber content of date seed in different species ranging from 2.7% to 8.23%.

**TABLE 1:** Approximate analysis and functional characteristics of quinoa flour and date seed powder.

Indexes	Samples	
	Quinoa flour	Date seed powder
Moisture (%)	6.32 ± 0.13	3.02 ± 0.39
Fat (%)	2.89 ± 0.66	10.18 ± 0.22
Protein (%)	12.97 ± 0.00	7.16 ± 0.06
Ash (%)	3.21 ± 0.21	0.88 ± 0.21
Fiber (%)	4.07 ± 0.02	18.52 ± 0.40
WHC (g/mL)	13.39 ± 0.39	13.15 ± 0.24
WHO (g/mL)	2.25 ± 0.13	1.51 ± 0.05

Functional properties such as water holding capacity (WHC) and oil holding capacity (OHC) are important physical characteristics that influence the quality of food products. Water holding capacity is influenced by the protein and fiber content of samples Kinsella (1979). It is noteworthy that the functional properties of date seed powder samples may vary depending on the content of soluble and insoluble fiber present in them Bouaziz et al. (2020). Also, differences in functional properties among different samples may be attributed to changes in fiber particle size, chemical composition, and the variety of dates used in the study Bouaziz et al. (2020). Additionally, spatial arrangement, hydrophilicity, hydrophobicity of proteins, and the presence of hydrophilic carbohydrates are also important factors in water holding capacity Seena and Sridhar (2005). In the current study, the water holding capacity of quinoa flour and date seed powder was determined to be 13.39 and 13.15 g/mL, respectively, while Liu et al. (2020) reported the water holding capacity of quinoa flour in the range of 1.2–7.3 g/mL. Furthermore, Jahan et al. (2023) reported an oil holding capacity of 2.56 g for date seed powder, which may vary among different species. It is also reported that the amount of water absorbed by food systems is closely related to the structure of compounds present in them, the number of polar groups, spatial arrangement, hydrophobicity, pH, temperature, ion strength, and protein concentration of the product Taghizadeh, Akhoondzadeh, and Zamani (2021).

Oil absorption is a physical phenomenon defined as the physical entrapment of oil, attributing it to non‐polar chains of its components and spatial protein structures Kaur, Kumar, and Bhat (2015). On the other hand, the hydrophobic compounds present in date seed powder are the main reason for their oil absorption properties Bouaziz et al. (2020). Moreover, this characteristic is related to the chemical composition, nature, and structure of the fiber, and the fiber molecule's affinity for oil Abbès et al. (2011). High oil absorption capacity is an important parameter that contributes to preserving aroma and flavor in food products and improving emulsifying properties Taghizadeh, Akhoondzadeh, and Zamani (2021). The current study revealed an oil holding capacity of 2.25 g/mL for quinoa flour. In this regard, Liu et al. (2020) reported the oil holding capacity of quinoa flour about 1.85–2.95 g/mL. Additionally, the oil holding capacity of date seed powder was determined to be 1.51 g/mL in the present study, which was higher than the reported value (31.1) by Jahan et al. (2023).

### 3.2 Approximate Composition of Chicken and Fish Nuggets

#### 3.2.1 Moisture Content

The results of Table 2 showed that the addition of fiber led to a partial decrease in the moisture content of chicken and fish nuggets, which can be attributed to the lower moisture content of quinoa flour (6.32%) and date seed powder (3.02%) compared to chicken and fish. As evident from the results, the highest moisture content of chicken and fish nuggets was observed in Treatments 1, with values of 67.8% and 72.4%, respectively (*p* < 0.05). Although the high fiber content of date seed powder increased moisture absorption by forming hydrogen bonds with water molecules in fried food and reduced water displacement by oil (Ammar 2017), replacing it with up to 3% could not compensate for the high moisture content of chicken and fish in the present study. A similar study showed that adding orange pulp powder and eggplant pulp powder as fiber sources reduced the moisture of the samples, which was in line with the present study (Ammar 2017). Additionally, the results obtained for fish nuggets were in line with the findings by Silva and Silva (2019), where adding different amounts of rice flour to croaker nuggets had an impact. It was found that adding 0%, 15%, and 30% rice flour to croaker nuggets revealed a significant difference in moisture content between the 30% rice flour treatment and the control treatment, with the low moisture content of rice flour being cited as the reason. Generally, it has been reported that the water absorption and moisture retention properties of meat products increase with increased fiber content. This is due to the high content of carboxyl groups, which can easily attract water molecules (Gutiérrez‐Silva et al. 2023).

**TABLE 2:** The effect of quinoa flour and date seed powder on the chemical properties of the produced chicken nuggets.

Indexes	Treatments			
	T1	T2	T3	T4
Chicken nuggets				
Moisture (%)	67/80 ± 0/34^a^	64/15 ± 0/80^b^	63/57 ± 1/21^b^	63/55 ± 0/41^b^
Fat (%)	6/07 ± 0/01^c^	6/64 ± 0/35^b^	6/63 ± 0/07^b^	7/25 ± 0/10^a^
Protein (%)	17/92 ± 0/18^a^	17/38 ± 0/35^a^	14/37 ± 0/82^b^	14/79 ± 0/34^b^
Ash (%)	2/30 ± 0/02^b^	2/57 ± 0/06^a^	2/61 ± 0/09^a^	2/61 ± 0/03^a^
Fiber (%)	0/94 ± 0/05^c^	1/41 ± 0/06^b^	1/45 ± 0/08^b^	2/16 ± 0/03^a^
Fish nuggets				
Moisture (%)	72/40 ± 0/22^a^	68/28 ± 0/39^b^	67/14 ± 0/62^c^	66/91 ± 0/22^c^
Fat (%)	6/94 ± 0/13^bc^	6/78 ± 0/17^c^	7/23 ± 0/11^ab^	7/54 ± 0/15^a^
Protein (%)	16/77 ± 0/28^a^	16/39 ± 0/10^b^	16/22 ± 0/01^b^	15/94 ± 0/11^c^
Ash (%)	1/70 ± 0/03^b^	1/87 ± 0/05^a^	1/77 ± 0/02^b^	1/73 ± 0/03^b^
Fiber (%)	1/68 ± 0/04^d^	1/92 ± 0/04^c^	2/12 ± 0/05^b^	2/39 ± 0/02^a^

*Note:* Treatment 1: Nugget emulsion without quinoa flour and date seed powder. Treatment 2: Nugget emulsion containing 6% quinoa flour. Treatment 3: Nugget emulsion containing 4.5% quinoa flour and 1.5% date seed powder. Treatment 4: Nugget emulsion containing 3% quinoa flour and 3% date seed powder. The average ± standard error values, common letters indicate no significant difference, and different letters indicate a significant difference in each column.

#### 3.2.2 Fat Content of Nuggets

The fat content results for chicken and fish nuggets are shown in Table 2. Among the chicken and fish nuggets, the highest fat content was observed in Treatment 4 (containing 3% quinoa flour and 3% date seed powder) with a value of 7.25% and 7.54% for chicken and fish nuggets, respectively (*p* < 0.05), which can be attributed to the high fat content of date seed powder. Similar results were seen in a study by Kaur, Kumar, and Bhat (2015) using pomegranate seed powder and tomato powder for chicken nugget production. These researchers attributed the significant increase in chicken nugget fat content with pomegranate seed powder to the presence of fatty acids such as puninic acid, linoleic acid, oleic acid, palmitic acid, stearic acid, and non‐steroidal phytochemicals present in pomegranate seeds.

#### 3.2.3 Protein Content of Nuggets

The protein analysis results (Table 2) in chicken nuggets showed a decreasing trend in the treated samples, indicating that with an increase in the percentage of quinoa flour and date seed powder, the protein content of chicken nuggets decreased from 17.38% to 14.79% compared to the control samples (17.92%). Similarly, in the produced fish nuggets, the control treatment exhibited the highest protein content with a significant difference compared to other treatments. The slight decrease in protein content in treatments containing quinoa flour and date seed powder compared to the control sample can be attributed to the higher protein content of chicken and fish compared to quinoa flour. This reduction is also because we used quinoa flour and date pit powder instead of 6% of the meat in the formulation. Since the protein content of date pit powder is even lower than quinoa, the fish nuggets containing 3% date pit powder and 3% quinoa flour have the lowest protein content. In this regard, Yadav et al. (2018) observed a slight decrease in protein content in chicken sausage with an increase in wheat bran level; they also reported a decrease in protein content by adding dried carrot powder compared to the control sample. In a study examining the effect of chickpea skin flour on the quality of low‐fat chicken nuggets, the results showed that adding chickpea skin flour up to 10% led to a gradual decrease in protein content, and there was a significant difference in protein content between treatments containing chickpea skin flour and the control treatment Verma, Banerjee, and Sharma (2012).

#### 3.2.4 Ash Content of Nuggets

The ash measurement results indicated that the highest ash content was present in formulated chicken and fish nuggets with fiber (Table 2). In the current study, the ash content increased after adding fiber, which was due to the higher ash content of quinoa flour and date seed powder compared to chicken meat (1%–1.5%) (Yadav et al. 2018). In a study by Yasarlar, Daglioglu, and Yilmaz (2007), the effects of adding cereal bran on the chemical composition, cooking properties, and sensory properties of Turkish meatballs showed that the ash content of the samples was significantly affected by the addition of bran. The results related to fish nuggets showed that Treatment 2, which only contained quinoa, had a higher ash content than the others and showed a significant difference compared to other treatments, while no significant difference was observed among the other treatments (*p* > 0.05). Biswas, Kandasamy, and Das (2022) demonstrated that by increasing the amount of dragon fruit peel powder in fish nuggets, the ash content also increased to the extent that three treatments containing dragon fruit peel powder differed significantly from the control treatment (without dragon fruit peel powder). In contrast to our findings, Binti Mohd Zaini et al. (2021) observed a decrease in ash content of low‐fat chicken nuggets when green peas were added at various levels, compared to control samples.

#### 3.2.5 Fiber Content of Nuggets

The fiber content evaluation results showed an increasing trend in samples formulated with quinoa flour and date seed powder (Table 2). The highest fiber content was observed in Treatment 4 (containing 3% quinoa flour and 3% date seed powder) with a value of 2.16%, which had a significant difference compared to other treatments (*p* < 0.05). Additionally, the analysis of fiber content in fish nuggets showed that adding quinoa flour and then date seed powder to the control nuggets demonstrated an increasing trend in fiber content, with Treatment 4 showing the highest amount (*p* < 0.05). The reason for this increase can be attributed to the higher fiber content of date seed powder. According to result, the fiber content of date seed powder is approximately 4.5 times that of quinoa flour. Therefore, Treatment 4, with the highest percentage of date seed powder, had the highest fiber content compared to other treatments. In line with this, a study was conducted with the aim of adding chickpea skin flour, which is high in fiber, to chicken nuggets. The results showed a significant increase in fiber content in the formulated chicken nuggets with chickpea skin flour compared to the control samples Verma, Banerjee, and Sharma (2012). In another study, by adding green peas to the formulation of chicken nuggets, a significant and remarkable increase in dietary fiber content was observed Binti Mohd Zaini et al. (2021).

### 3.3 Physicochemical Characteristics of Chicken and Fish Nuggets

The effect of adding different fiber sources on the physical properties of chicken and fish nuggets is shown in Table 3, respectively.

**TABLE 3:** The effect of addition of quinoa flour and date seed powder on the physicochemical properties of chicken and fish nuggets.

Treatments	Indexes				
	pH emulsion	pH product	WHC (%)	Cooking yield (%)	ES (%)
Chicken nuggets					
T1	7/27 ± 0/07^c^	7/33 ± 0/05^c^	81/10 ± 0/97^b^	86/23 ± 0/24^c^	90/70 ± 0/42^c^
T2	7/63 ± 0/05^a^	7/70 ± 0/00^a^	86/30 ± 0/39^a^	90/17 ± 0/77^b^	94/72 ± 0/45^b^
T3	7/47 ± 0/05^b^	7/47 ± 0/05^b^	87/74 ± 0/54^a^	93/03 ± 0/72^a^	96/08 ± 0/82^ab^
T4	7/40 ± 0/01^b^	7/47 ± 0/05^b^	86/95 ± 0/58^a^	91/40 ± 1/70^ab^	96/50 ± 0/74^a^
Fish nuggets					
T1	7/49 ± 0/07^b^	7/67 ± 0/03^a^	77/51 ± 1/84^b^	87/07 ± 0/85^b^	97/71 ± 0/20^a^
T2	7/64 ± 0/04^a^	7/68 ± 0/03^a^	84/84 ± 1/48^a^	87/52 ± 1/57^b^	98/38 ± 0/26^b^
T3	7/41 ± 0/07^b^	7/45 ± 0/01^c^	85/37 ± 0/43^a^	91/27 ± 1/49^a^	98/52 ± 0/18^b^
T4	7/48 ± 0/03^b^	7/55 ± 0/06^b^	85/48 ± 1/84^a^	88/06 ± 0/75^b^	98/81 ± 0/74^b^

*Note:* Treatment 1: Nugget emulsion without quinoa flour and date seed powder. Treatment 2: Nugget emulsion containing 6% quinoa flour. Treatment 3: Nugget emulsion containing 4.5% quinoa flour and 1.5% date seed powder. Treatment 4: Nugget emulsion containing 3% quinoa flour and 3% date seed powder. The average ± standard error values, common letters indicate no significant difference, and different letters indicate a significant difference in each column.

#### 3.3.1 pH Emulsions and Products

pH is an important parameter that indicates the functional characteristics and quality preservation of meat during storage. The pH of muscle decreases after slaughter due to the production of lactic acid, reaching a final level of 5.5–5.7 (Talukder 2015). In the current study, the pH of the emulsion produced was in the range of 6.63–6.27. The data in Table 3 showed that there was a significant difference between the control nuggets and the fiber‐containing treatments in terms of pH values (*p* < 0.05). This trend was observed not only in the emulsion (raw nuggets) but also in the fried nuggets. Additionally, the pH of the produced products ranged from 6.33 to 6.70. The pH values of the fried chicken nuggets were higher than the raw samples, and this slight increase in pH can be attributed to the release of alkaline compounds from amino acids after heating, increased salt concentration due to moisture loss, and changes in protein net charge due to denaturation, which was consistent with the results of Ammar (2017b). Interestingly, similar results were observed for the change in pH of chicken nuggets before and after cooking for fish nuggets (Table 3). The evaluation of the quality of chicken nuggets containing shredded carrots and sweet potato puree showed that adding these two ingredients to the nugget formulation significantly affected the pH of both raw and cooked products. The decrease in emulsion pH can be attributed to the higher acidity of the added vegetables in the products, but overall, the pH of the fried samples was higher than the emulsion samples, which can be related to protein denaturation and the release of free amino groups, especially SH groups at higher temperatures (Bhosale et al. 2011). Biswas and Nanda (2023) also reported an increase in acidity in fish nuggets due to the addition of guava peel powder, attributed to its rich content of ascorbic acid.

#### 3.3.2 Water Holding Capacity of Nuggets

Water holding capacity (WHC) is one of the most important characteristics of meat emulsion products, as it reflects the amount of water retained by a product under external forces (Basharat et al. 2023). As evident from the results, the highest WHC value was observed in Treatment 3 with 87.74%, which had a significant difference compared to Treatment 1 (control) (*p* ≤ 0.05) (Table 3). It is worth mentioning that no significant difference was observed between the other treatments (*p* ≥ 0.05). The data from the current study indicated that the water holding capacity of raw or cooked chicken meat nuggets increases with an increase in dietary fiber content. As discussed in the section regarding the water absorption properties of quinoa flour and date pit powder fibers, one of the reasons for the increased water holding capacity of nuggets containing quinoa flour and date pit powder compared to the control sample can be attributed to the fibers' ability to bind to water and fats (Pathera et al. 2017). Furthermore, the carboxyl groups present within the fiber structure are highly effective in binding and retaining water. Similar results were obtained by Talukder and Sharma (2010), where the water holding capacity of chicken nuggets increased with an increase in dietary fiber content. Also, Table 3 showed the water holding capacity of fish nuggets in this study ranged from 77.51% to 85.48%. The lowest water holding capacity was in the control treatment with 77.51%, and the highest moisture content was in Treatment 4 with 88.48%. This significant increase in water holding capacity may be due to the specific water holding properties of date seed powder and quinoa flour. In line with this, a study on the effect of adding various levels of banana peel powder (0%, 2%, 4%, and 6%) to fish cutlets by Binti Mohd Zaini et al. (2021) found that the addition of banana peel powder significantly increased the water holding capacity of the fish cutlets, which aligns with the results of our study. Additionally, Xu et al. (2018) reported a significant increase in water holding capacity by adding corn flour and sago flour to fish nuggets compared to the control treatment. The researchers attributed this to the presence of amylose and amylopectin in corn and sago flour, which create a matrix that can hold the most water, ultimately increasing the water holding capacity.

#### 3.3.3 Cooking Yield of Nuggets

Cooking yield is an important indicator in the evaluation of breaded and fried products, as it has a direct relationship with the final product weight and is economically significant Heydari, Shabanpour, and Pourashouri (2018). In the present work, the product yield increased with an increase in fiber content due to water retention properties compared to the control (Table 3). The highest yield was related to Treatment 3 (93.03%) with the highest water holding capacity. Additionally, the presence of fiber in meat products leads to a reduction in cooking loss Verma, Rajkumar, and Kumar (2019). The significant increase in cooking yield in treatments containing fiber compared to the control is likely due to the reduction in shrinkage during cooking attributed to the fiber (Kumar et al. 2013). This can be attributed to the reduced oil absorption and increased moisture retention by the fiber present in quinoa flour and date pit powder during the frying process. The fiber in quinoa flour and date pit powder contributes to lower oil uptake and higher moisture absorption in the fried food. This is due to the hydrogen bonding between water molecules and the fiber, which prevents the displacement of water by frying oil (Fiszman and Salvador 2003; Kim et al. 2015). Furthermore, it is unclear how the addition of fiber affects the dough pick‐up in chicken nuggets, but it appears that fiber influences the adhesive properties of chicken nuggets, which may explain the higher baking yield observed in the fiber‐containing nuggets (Alrawashdeha and Abu‐Alruza 2022; Fiszman and Salvador 2003). Different types of dietary fiber (alone or in combination) can be used to improve the cooking performance and texture of meat products (Kim et al. 2015). In a study using various cereal brans (oat, corn, barley, and wheat) as dietary fiber sources in the production of meatballs, the results showed that the control meatballs had the highest cooking loss and the lowest yield, which was consistent with the current study. Other studies have also obtained similar results, including the addition of orange pulp powder and eggplant peel powder in chicken nugget formulation at additive levels of 5% and 10%, resulting in increased product yield (Ammar 2017). Table 3 also shows the cooking yield of fish nuggets in this study ranging from 87.07% to 91.27%. The lowest cooking yield was in the control treatment, and the highest yield was in Treatment 4, which contained equal proportions of quinoa flour and date seed powder (*p* < 0.05). In a study by Binti Mohd Zaini et al. (2021) that examined the cooking yield of fish cutlets with the addition of banana peel powder, it was reported that adding dietary fiber to the fish cutlets increased the cooking yield. Researchers attributed the higher cooking yield to the higher water holding capacity of the fish cutlets containing dietary fiber. The presence of dietary fiber increases the water holding capacity, allowing water to occupy the insoluble fiber pores such as polysaccharides connected by hydrogen or ionic bonds, leading to improved cooking performance through surface tension and increased product stability during freezing (Banerjee et al. 2020; Biswas and Nanda 2023).

#### 3.3.4 Emulsion Stability of Chicken and Fish Nuggets

The effect of adding quinoa flour and date seed powder on the stability of the treatments' emulsions is shown in Table 3. The highest emulsion stability was observed in Treatment 4 (96.50%) with the highest fiber content, while the lowest stability was in the control sample (90.70%) (*p* < 0.05). The increase in emulsion stability in chicken nuggets with an increase in fiber percentage is due to the moisture trapping in the form of gel during the application of heat Kaur, Kumar, and Bhat (2015). In line with this, previous research has demonstrated the increase in emulsion stability of samples by adding green banana flour and soybean hull flour as fiber sources (Kumar et al. 2013). The significant increase in emulsion stability and yield of these treatments can be attributed to the increase in viscosity by fiber. In a study by Kaur, Kumar, and Bhat (2015), a significant increase in emulsion stability in chicken nuggets with tomato powder combination was observed. The increase in emulsion stability can also be linked to the increase in starch gelatinization properties during heating, which stabilizes the emulsion. Based on the mentioned studies, the presence of fiber sources in meat products increases water retention properties, leading to improved emulsion stability. Table 3 also shows the emulsion stability of fish nuggets in this study ranging from 97.71% to 98.81%. Biswas, Kandasamy, and Das (2022) stated that by adding dragon fruit peel powder to fish nuggets, the emulsion stability of the fish nuggets increased significantly due to the presence of dietary fiber and, consequently, high water holding capacity. The emulsion stability of fish nuggets containing guava peel powder was significantly higher than the control treatment. Researchers attributed this to the presence of dietary fiber, phytochemical properties, antioxidants, carotenoids, and phenolics in guava peel powder (Biswas and Nanda 2023). However, fiber addition to nuggets has been shown to reduce emulsion stability in some studies. For instance, using pomegranate seed powder and tomato powder in the production of fiber‐enriched chicken nuggets demonstrated a significant decrease in emulsion stability with increasing levels of pomegranate seed powder Kaur, Kumar, and Bhat (2015). This may be attributed to a decrease in pH, leading to a reduction in the corresponding ratio. These findings could be due to the presence of a higher amount of soluble dietary fiber, which traps and holds moisture in a gel form during heat application Kaur, Kumar, and Bhat (2015); Mehta et al. (2015).

#### 3.3.5 Oil Absorption

Oil absorption depends on factors such as oil quality, frying time, oil temperature, shape, porosity, food composition, especially initial water content, surface roughness of the coated product, and other factors (Xie et al. 2022). Additionally, the oil absorption in fried foods was significantly influenced by their microstructure and surface traits, based on three suggested mechanisms: water replacement, a cooling phase effect, and the surfactant theory of frying (Shan et al. 2018). As observed (Table 4), the lowest oil absorption in chicken nuggets was related to Treatment 4 (3.82%), showing a significant statistical difference at the 5% level compared to other treatments. Additionally, the lowest oil absorption in fish nuggets was related to Treatment 4 (*p* < 0.05). The formation of hydrogen bonds between water and fiber molecules and prevention of water substitution by oil lead to reduced oil absorption. Additionally, the carboxylic (–COOH) and hydroxyl (–OH) groups, along with other hydrophilic groups found in the fiber sources utilized in this study, can form hydrogen bonds with water in both the crust and core, effectively reducing moisture evaporation in those areas (Shan et al. 2018). Similar results were observed in a study by Kim et al. (2015) in the use of wheat fiber in chicken nugget formulation and in the effect of adding cellulose powder to reduce oil absorption. Similar trends in cooking performance were also reported in studies related to adding dietary fibers to meat products (Choi et al. 2009). Furthermore, studies indicate that the evaporation rate of moisture from the crust and core increased considerably during deep‐fat frying, resulting in the development of large cavities in the crust. This process created a very low positive vapor pressure, which aided in the oil absorption of the battered and breaded fish nuggets during both the frying and cooling (Shan et al. 2018). Nevertheless, incorporating higher levels of fiber into the nugget recipe can decrease their evaporation rate by retaining water molecules, thereby reducing oil absorption naturally.

**TABLE 4:** The effect of quinoa flour and date seed powder on the reduction of oil absorption of chicken and fish nuggets in terms of grams per 100 g of sample.

Samples	Treatments			
	T1	T2	T3	T4
Chicken nuggets	4/74 ± 0/10^a^	4/68 ± 0/28^a^	4/49 ± 0/43^a^	3/82 ± 0/29^b^
Fish nuggets	7/56 ± 0/33^b^	7/48 ± 0/23^a^	6/55 ± 0/40^b^	5/19 ± 0/44^c^

*Note:* The average ± standard error values, common letters indicate no significant difference, and different letters indicate a significant difference in each column.

### 3.4 The Effect of Quinoa Flour and Date Seed Powder on the Color Characteristics of the Produced Nuggets

The results of the color analysis of chicken and fish nugget samples are presented in Table 5. In both chicken and fish nugget treatments, the highest brightness index values (45.80 in chicken nuggets and 41.60 in fish nuggets) were associated with Treatment 2, which had the highest amount of quinoa flour, while the lowest values were observed in Treatment 4, which contained the highest amount of date seed powder. The brown pigments present in date seed powder led to a decrease in the L* index in the samples. Additionally, the increase in the a* index in chicken and fish nugget samples is likely due to the increase in pigments present in date seed powder. In terms of the effects of additives and food components on product color quality, the decrease in brightness index resulting from the effect of crown flower and quinoa grain in goat meat nuggets Verma, Rajkumar, and Kumar (2019) and okara flour and rice bran in chicken nuggets can be noted Echeverria et al. (2022). In a study by Binti Mohd Zaini et al. (2021), a decreasing trend in L* and a* values and an increase in b* values for samples treated with green peas were observed, showing a significant difference compared to the control treatment. These results were attributed to the antioxidant changes in green peas and their impact on the final product color. Additionally, in another study, the effect of pomegranate peel powder on fried chicken indicated a significant impact of this additive on the product color Basharat et al. (2023). However, in a study replacing animal fat with pumpkin flour in meatballs did not significantly alter the lightness (*L* value) but resulted in significant differences in redness (*a* value) and yellowness (*b* value) Saleh et al. (2014). They stated that as pumpkin flour content increased, *a* value decreased and *b* values increased, indicating a shift towards a more yellowish color, likely attributable to the natural pigmentation of pumpkin flour.

**TABLE 5:** The effect of quinoa flour and date seed powder on the chemical properties of the produced chicken nuggets.

Indexes	Treatments			
	T1	T2	T3	T4
Chicken nuggets				
L*	45/50 ± 2/75^a^	45/80 ± 2/78^a^	44/50 ± 3/65^a^	41/10 ± 0/80^b^
a*	4/80 ± 1/98^bc^	3/70 ± 1/15^c^	5/70 ± 1/82^ab^	6/90 ± 2/02^a^
b*	40/30 ± 2/75^a^	37/30 ± 2/00^ab^	35/70 ± 4/21^b^	39/70 ± 3/43^a^
Fish nuggets				
L*	37/20 ± 2/14^ab^	41/60 ± 5/57^a^	33/00 ± 2/29^bc^	27/60 ± 3/88^c^
a*	8/20 ± 0/98^a^	10/00 ± 1/26^a^	10/00 ± 1/79^a^	9/60 ± 1/20^a^
b*	31/40 ± 4/76^a^	23/60 ± 1/62^b^	26/00 ± 1/79^b^	22/00 ± 0/63^b^

*Note:* Treatment 1: Nugget emulsion without quinoa flour and date seed powder. Treatment 2: Nugget emulsion containing 6% quinoa flour. Treatment 3: Nugget emulsion containing 4.5% quinoa flour and 1.5% date seed powder. Treatment 4: Nugget emulsion containing 3% quinoa flour and 3% date seed powder. The average ± standard error values, common letters indicate no significant difference, and different letters indicate a significant difference in each column.

### 3.5 The Effect of Quinoa Flour and Date Seed Powder on the Textural Properties of the Produced Nuggets

The properties related to the texture index of chicken and fish nugget samples containing quinoa flour and date seed powder, including hardness, gumminess, chewiness, and springiness, are shown in Table 6, respectively. The results indicate that in both chicken and fish nuggets, the highest hardness values were associated with Treatment 4, while the lowest hardness values were observed in the control treatment. All treatments had a significant difference at the 5% level. The significant increase in hardness in Treatment 4 of both chicken and fish nuggets can be attributed to the higher fiber content in these treatments. Previous research has shown that as the moisture content of food decreases, its hardness increases, with the lowest hardness values observed in products with the highest moisture content. Generally, an inverse relationship is seen between the moisture content of food and its hardness index (Das et al. 2008). In a study examining the effect of different mixtures of chicken skin and wheat fiber on the properties of chicken nuggets, treatments containing fiber showed higher hardness characteristics compared to the control treatment (Kim et al. 2015). Additionally, the study adding green banana flour and soy husk to chicken nugget formulation showed significant differences in the firmness of the samples with added flour compared to the control treatment (Kumar et al. 2013). These researchers attributed the higher hardness of these treatments to the chemical composition of these flours and their ability to create a firmer texture. In the mentioned study, the gumminess, chewiness, and springiness values of the control samples were lower compared to the formulated products. These results were consistent with the results of our study, where treatments formulated with quinoa flour and date seed powder showed higher values in texture indices. Furthermore, adding date seed powder to veal and lamb resulted in a significant increase in the gumminess property of the final cooked product (Nor et al. 2022). An increase in fiber content may lead to slight changes in the texture parameters of the product due to increased water and fat binding capabilities (Ammar 2017), or depending on the type of flour, it may result in a noticeable decrease in hardness, springiness, gumminess, and chewiness of the nuggets. In general, reducing the amount of meat in protein products due to a decrease in protein content has a significant impact on water binding, structural integrity, and gelation of the product. Therefore, adding plant fiber to a meat product may be beneficial for the desired texture Oshodi, Ogungbenle, and Oladimeji (1999).

**TABLE 6:** The effect of addition of quinoa flour and date seed powder on the physicochemical properties of chicken and fish nuggets.

Treatments	Indexes			
	Hardness (*N*)	Gumminess (*N*)	Chewiness (*N*)	Springiness (mm)
Chicken nuggets				
T1	2/51 ± 0/29^c^	1/59 ± 0/18^b^	1/36 ± 0/34^a^	0/71 ± 0/01^b^
T2	2/88 ± 0/38^bc^	2/01 ± 0/14^ab^	1/63 ± 0/02^a^	0/75 ± 0/02^a^
T3	3/27 ± 0/30^ab^	2/19 ± 0/16^a^	1/64 ± 0/95^a^	0/75 ± 0/01^a^
T4	3/62 ± 0/53^a^	2/30 ± 0/47^a^	1/75 ± 0/26^a^	0/76 ± 0/02^a^
Fish nuggets				
T1	4/13 ± 0/38^b^	2/23 ± 0/25^b^	1/60 ± 0/60^a^	0/79 ± 0/03^c^
T2	5/82 ± 0/66^c^	3/43 ± 0/33^a^	1/94 ± 0/44^a^	0/84 ± 0/02^b^
T3	6/44 ± 0/48^a^	3/46 ± 0/13^a^	1/94 ± 0/60^a^	0/91 ± 0/02^a^
T4	6/36 ± 0/47^a^	3/18 ± 0/10^a^	2/03 ± 0/62^a^	0/91 ± 0/02^a^

*Note:* Treatment 1: Nugget emulsion without quinoa flour and date seed powder. Treatment 2: Nugget emulsion containing 6% quinoa flour. Treatment 3: Nugget emulsion containing 4.5% quinoa flour and 1.5% date seed powder. Treatment 4: Nugget emulsion containing 3% quinoa flour and 3% date seed powder. The average ± standard error values, common letters indicate no significant difference, and different letters indicate a significant difference in each column.

### 3.6 The Effect of Quinoa Flour and Date Seed Powder on the Sensory Properties of the Produced Nuggets

**FIGURE 2:**
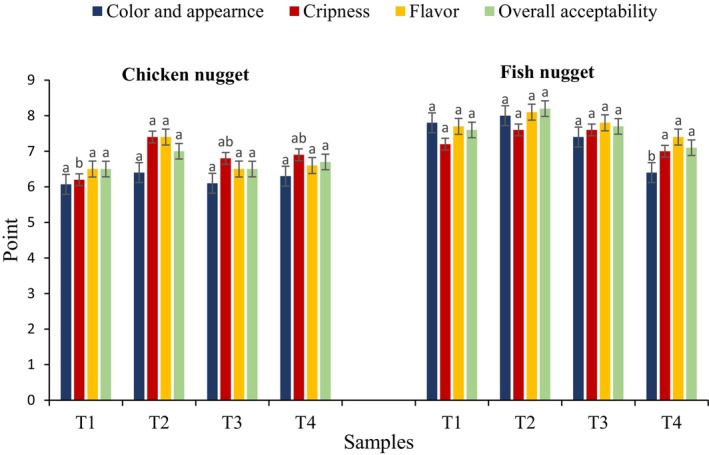
The effect of quinoa flour and date seed powder on the sensory properties of the produced nuggets.

The sensory evaluation of fried chicken and fish nugget samples with four different formulations using a 9‐point hedonic scale is presented in Figure 1. The results of the current study indicated that although there were differences in the scores obtained for color and appearance, taste, and overall acceptability of the produced nuggets, these differences were not significant (*p* ≥ 0.05). In terms of color and appearance, the formulated chicken and fish nuggets with quinoa flour and date seed powder did not differ significantly from the control samples (*p* ≥ 0.05), although Treatment 2 with the highest quinoa content had a higher color score (6.40). Color, as a visual phenomenon, is one of the fundamental factors in determining the quality and acceptance of a product. The crispness, taste, and appearance scores of the formulation treated with quinoa flour (Treatment 2) were higher than the other treatments, resulting in higher overall acceptability. The sensory evaluation results of fish nuggets are shown in Figure 2, where it is evident that the only index where a significant difference was observed among the treatments was the color and appearance index. Treatment 2, which contained 6% quinoa, scored the highest in this index, while in other sensory evaluation indices such as tenderness, taste, and overall acceptability, this treatment, despite having the highest score, did not show a significant difference from the other treatments (*p* > 0.05). A study by El‐Sohaimy et al. (2022) showed that chicken nuggets coated with quinoa flour did not have a significant difference from the control samples in all sensory criteria, including color and appearance, tenderness, and taste, although their overall acceptability differed. The sensory evaluation by these researchers indicated that substituting wheat flour with quinoa flour in nuggets had no significant impact on the organoleptic properties and consumer acceptance of the product. Another study aiming to replace soy flour with quinoa flour in beef burger production showed no significant differences in color, taste, and aroma between the burger treatments, but a significant difference was observed in the texture and juiciness index, with quinoa flour‐treated groups showing better sensory acceptance, especially in texture and juiciness, without negatively affecting the sensory properties of the beef burger (Shokry 2016). Summary, adding dietary fiber to meat products affects sensory properties depending on its concentration, and dilution of meat taste with higher levels of fiber sources may lead to a reduction in taste scores of fiber‐enriched meat products.

## 4 Conclusions

This study investigated the potential of date seed powder and quinoa flour as fiber sources to enhance the nutritional and functional properties of chicken and fish nuggets. Our findings demonstrate that incorporating these flours effectively increased the fiber content of both types of nuggets, significantly improving their nutritional value. Notably, the addition of these flours had a positive impact on the physicochemical properties of the nuggets, particularly in terms of texture and color. While the sensory evaluation of chicken nuggets showed no significant changes in most aspects except for color and appearance, fish nuggets exhibited a significant difference in tenderness. These results suggest that date seed powder and quinoa flour can be valuable additions to the formulation of processed meat products, offering a means to enhance their nutritional profile and potentially improve their texture and color. Further research could explore the optimal ratios of these flours to achieve desirable sensory attributes and explore the potential applications in other meat‐based products.

## Author Contributions

**Shahab Naghdi:** investigation, methodology, formal analysis, writing – original draft. **Masoud Rezaei:** supervision, project administration, conceptualization, resources, writing – review & editing. **Mahboobeh Kashiri:** conceptualization (equal), formal analysis (equal), supervision (equal), validation (equal). **Fatemeh Rezaei:** investigation (equal), methodology (equal), writing – original draft (equal). **Serva Naseri:** investigation (equal), methodology (equal). **Hossein Nourani:** formal analysis (equal), investigation (equal), methodology (equal). **Zahra Khakpour:** formal analysis (equal), investigation (equal), methodology (equal).

## Ethics Statement

The authors have nothing to report.

## Conflicts of Interest

The authors declare no conflicts of interest.

## Data Availability

The data that support the findings of this study are available from the corresponding author upon reasonable request.
